# Geometry and evolution of Hasimoto surface in Minkowski 3-space

**DOI:** 10.1371/journal.pone.0294310

**Published:** 2024-01-02

**Authors:** H. S. Abdel-Aziz, H. M. Serry, F. M. El-Adawy, M. Khalifa Saad

**Affiliations:** 1 Department of Mathematics, Faculty of Science, Sohag University, Sohag, Egypt; 2 Department of Mathematics, Faculty of Science, Suez Canal University, Ismailia, Egypt; 3 Department of Mathematics, Faculty of Science, Islamic University of Madinah, Madinah, KSA; Tel Aviv University, ISRAEL

## Abstract

The main goal of this paper is to introduce the evolution equations for a timelike Hasimoto surface from its fundamental form coefficients in Minkowski 3-space $E_{1}^{3}$. By utilizing the evolved quasi-curve (q-curve), we present and analyze three types of Hasimoto surfaces, attributed to the quasi-tangent, quasi-normal, and quasi-binormal vectors of the curve. Finally, we provide an illustrated example to strengthen our main results.

## Introduction

A key topic that has previously been explored in many different domains is the phenomenon of how to produce the evolution of curves or surfaces. Flows, and more specifically the inextensible flows that might occur along a curve or surface, are what cause the time evolution of a curve or surface. We will refer to curves evolution also as flows. If the arc length or the intrinsic curvature of a surface is preserved throughout the flow of curve or surface, respectively, we then say that the flow is inextensible [1–3]. Several scholars have researched geometric flow issues on curves and surfaces in different spaces in recent years (see for instance [1, 4–6]). At any point on a curve, there are many frames associated with it.

In this paper, we use the quasi-frame due to its importance and ease of application compared to the other available frames. For example, the frame of Frenet is not described when the curvature disappears. Aside from that, the Frenet framework’s biggest limitation is its undesirable tangent vector rotation has further information, [7, 8]. Bishop developed a new frame along a space curve that is more application-friendly, [9]. However, it is well known that computing Bishop frames is not simple task, as shown in [9, 10]. To address these issues, Coquillart [11] implemented the quasi-normal vector of a space curve to create the 3D curve offset. At each point on a curve, a quasi-normal vector is defined, and it lies in the plane perpendicular to the curve’s tangent at that point. Compared to other frames such as Frenet and Bishop, the quasi-frame has several advantages. For example, the quasi-frame may be described along a line, and the creation of the quasi-frame is unaffected by whether the space curve has unit speed or not. Furthermore, the quasi-frame is conveniently determined [12].

In this work, we are interested in studying a Hasimoto surface which classified as one of the surfaces that can be described by integral equations. Such surfaces have a constant negative Gaussian curvature, as well as constant mean curvature, minimal surfaces, and affine spheres. Hasimoto surfaces provide a geometric representation of curves, especially spacelike and timelike curves, within Minkowski space. They offer an elegant way to visualize and study the behavior of curves in this non-Euclidean space [2]. Researchers have used Hasimoto surfaces to develop evolution equations that describe the change in shape and properties of these surfaces over time. These equations are instrumental in understanding the dynamics of curves in Minkowski space [13]. Hasimoto surfaces are employed in differential geometry to investigate the curvature and torsion properties of curves. They help in studying the relationship between various geometric quantities associated with curves.

A Hasimoto surface is the surface traced out by a curve *r* as it propagates in $E_{1}^{3}$ and evolves over time according to the evolution equation

$$\begin{array}{c} r_{t}=r_{s}\times r_{ss}, \end{array}$$ (1)

in other word,

$$\begin{array}{c} r_{t}=\kappa \text{p}. \end{array}$$ (2)

This is an evolution of the curve in its binormal direction with velocity equal to its curvature. Eqs (1) and (2), known as the vortex filament flow or smoke ring equations, and it may be thought of as a dynamical system on the space of curves in $E_{1}^{3}$ [14]. These equations were studied by Hasimoto [2], where refer to *r*(*s*, *t*) as the position vector for a point on the curve, where *s* is the arc length parameter, *t* is the time, *κ* and **p** are the curvature and the unit binormal vector of *r*, respectively.

The geometrical properties of solutions to the Eq (1) can be written as *r* = *r*(*s*, *t*). These properties represent our main aim. We can despite the geometric categorization of *r*(*s*, *t*) as follows:

(1) In the event that *r* = *r*(*s*, *t*) is a spacelike curve that includes a timelike normal vector field for every *t*, then the motion that satisfies Eq (1) will produce a spacelike Hasimoto surface.
(2) In the event that *r* = *r*(*s*, *t*) is a spacelike curve that includes a timelike binormal vector field for every *t*, then the motion that satisfies Eq (1) will produce a timelike Hasimoto surface.
(3) If *r* = *r*(*s*, *t*) is a timelike curve for every *t*, then the motion that creates a timelike Hasimoto surface is the motion that satisfies condition (1), see [14, 15].

Numerous spaces, including the Euclidean space [16], Minkowski space [17], Galilean space [18], and pseudo-Galilean space [5], have been used to study the equations of motion of curves and surfaces. Within the scope of our work, we investigate the evolution equations of Hasimoto surface by employing the quasi-frame of spacelike curve with timelike binormal. We begin by determining the equations of motion for the considered evolved curve via its quasi-frame and the velocity vector of that curve.

Throughout this paper, we assume that the tangent to the curve *r* is spacelike and the binormal is timelike, as in case (2). According to Hasimoto’s [2], the behavior of a thin vortex filament, thought of as a flowing space curve, could be translated to the nonlinear Schrodinger equation. The flowing curve of the sine Gordon equation was analyzed by Rick Mukherjee and Radha Balakrishnan [19]. In [5, 16], the authors investigated the motion of plane curves, hypersurface motion, and the motion of space curves in various spaces. By using the fundamental existence and uniqueness hypothesis of space curves, the authors in [13] developed Hasimoto surface via integration for Frenet-Serret equations.

Here, let’s employ a different strategy using a different approach. The main concept of this method is to construct the coefficients of the first and second fundamental forms of the Hasimoto surface, and then utilize the Gauss-Weingarten equations to determine their equations of motion by means of Christoffel symbols of the second type.

The paper is organized as follows: In Section: “Geometric preliminaries”, we provide a brief review of the geometry of curves, particularly spacelike curves related to our study of timelike Hasimoto surfaces. Section: “Evolution of spacelike q-curve” explores the evolution equations and various geometric properties of a timelike Hasimoto surface situated in Minkowski 3-space. To achieve this, we employ Gauss and Weingarten equations and explore changes occurring in the evolved q-curve associated with the Hasimoto surface under consideration. Additionally, we track the evolution of coefficients characterizing the surface’s first and second fundamental forms, as well as the Gaussian and mean curvatures, which are discussed in Section: “Geometry of Hasimoto surface”. To enhance our findings and provide a practical demonstration, we include a computational example in Section: “Application”. This example not only serves to illustrate our primary results but also features graphical representations for clarity.

## Geometric preliminaries of curves in $E_{1}^{3}$

In this section, we give a brief review of the geometry of curves in the Minkowski space needed in our study.

Minkowski space $E_{1}^{3}$ is the real vector space *E*^3^ expanded by the Lorentzian inner product

$$\begin{array}{c} {\left\langle \text{a},\text{b}\right\rangle }_{E_{1}^{3}}=a_{1}b_{1}-a_{2}b_{2}+a_{3}b_{3}, \end{array}$$ (3)

where **a** = (*a*_1_, *a*_2_, *a*_3_) and **b** = (*b*_1_, *b*_2_, *b*_3_) $\in E_{1}^{3}$. The norm of **b** is $\left||\text{b}|\right|=\sqrt{{\langle \text{b},\text{b}\rangle }_{E_{1}^{3}}}$.

Also, the cross product of **a** and **b** is referred to as

$$\begin{array}{c} \text{a}\;\wedge_{E_{1}^{3}}\;\text{b}=|\begin{array}{ccc} e_{1} & -e_{2} & e_{3}\\ a_{1} & a_{2} & a_{3}\\ b_{1} & b_{2} & b_{3} \end{array}| \end{array}$$

$$\begin{array}{c} =<-a_{3}b_{2}+a_{2}b_{3},-a_{3}b_{1}+a_{1}b_{3},a_{1}b_{2}-a_{2}b_{1}>. \end{array}$$ (4)

If $r\left(s\right):J\subseteq R\to E_{1}^{3}$ is a regular curve described this way

$$\begin{array}{c} r(s)=(y(s),z(s),w(s)), \end{array}$$ (5)

where J is an open interval and *y*(*s*), *z*(*s*)*andw*(*s*) ∈ *C*^3^. Such a curve is categorized as the following

1. Spacelike curve if ${\langle r^{\prime }\left(s\right),r^{\prime }\left(s\right)\rangle }_{E_{1}^{3}}>0$,
2. Timelike curve if ${\langle r^{\prime }\left(s\right),r^{\prime }\left(s\right)\rangle }_{E_{1}^{3}}<0$,
3. Lightlike curve if ${\langle r^{\prime }\left(s\right),r^{\prime }\left(s\right)\rangle }_{E_{1}^{3}}=0$, for all *s* ∈ *J*. The arc length parameter of the regular curve is defined as
  $$\begin{array}{c} l\left(s\right)=\int_{r}\sqrt{{\left\langle r^{\prime }\left(s\right),r^{\prime }\left(s\right)\right\rangle }_{E_{1}^{3}}}\;dt, \end{array}$$ (6)
  where the curve is said to be parameterized by the arc length when $\sqrt{{\langle r^{\prime }\left(t\right),r^{\prime }\left(t\right)\rangle }_{E_{1}^{3}}}=1$.

The trihedron frame of the curve with tangent **T**(*s*), principal normal **n**(*s*) and binormal **p**(*s*), takes the following structure:

$$\begin{array}{c} (\begin{array}{c} {\text{T}}_{s}\left(s\right)\\ {\text{n}}_{s}\left(s\right)\\ {\text{p}}_{s}\left(s\right) \end{array})=(\begin{array}{ccc} 0 & \kappa (s) & 0\\ \epsilon_{\text{p}}\kappa \left(s\right) & 0 & \tau (s)\\ 0 & \epsilon_{\text{T}}\tau \left(s\right) & 0 \end{array})(\begin{array}{c} \text{T}(s)\\ \text{n}(s)\\ \text{p}(s) \end{array}), \end{array}$$ (7)

where

$$\begin{array}{c} {\left\langle \text{T},\text{T}\right\rangle }_{E_{1}^{3}}=\epsilon_{\text{T}},\;\;\;\;\;\;\;\;{\left\langle \text{n},\text{n}\right\rangle }_{E_{1}^{3}}=\epsilon_{\text{n}},\;and\;{\left\langle \text{p},\text{p}\right\rangle }_{E_{1}^{3}}=-\epsilon_{\text{T}}\epsilon_{\text{n}}=\epsilon_{\text{p}}, \end{array}$$ (8)

and

$$\begin{array}{c} \text{T}\times_{E_{1}^{3}}\text{n}=\text{p},\;\;\text{n}\times_{E_{1}^{3}}\text{p}=-\epsilon_{\text{n}}\text{T},\;and\;\;\text{p}\times_{E_{1}^{3}}\text{T}=-\epsilon_{\text{T}}\text{n}. \end{array}$$ (9)

The functions *κ*(*s*) and *τ*(*s*) are the curvatures of the curve, for more details see [20].

We denote by {**T**_**q**_, **n**_**q**_, **P**_**q**_, **k**_**q**_} for the quasi-frame, and *r*^*q*^ parameterized by arc length *s* and

$$\begin{array}{c} {\text{T}}_{\text{q}}=\frac{r^{\prime q}}{\left|\right|r^{\prime q}\left|\right|},\;\;{\text{n}}_{\text{q}}=\frac{{\text{T}}_{\text{q}}\wedge_{E_{1}^{3}}k_{\text{q}}}{\left|\right|{\text{T}}_{\text{q}}\wedge_{E_{1}^{3}}k_{\text{q}}\left|\right|},\;\;{\text{p}}_{\text{q}}={\text{T}}_{\text{q}}\wedge_{E_{1}^{3}}{\text{n}}_{\text{q}}, \end{array}$$ (10)

where **T**_**q**_, **n**_**q**_, **p**_**q**_ and **k**_**q**_ represent the quasi-tangent, quasi-normal, quasi-binormal and the quasi-projection vectors, respectively [21]. The quasi-projection vector is sometimes selected with varying values like **k**_**q**_ = (0, 1, 0) (spacelike) or **k**_**q**_ = (1, 0, 0) (spacelike) and in this sense, both quasi-tangent **T**_**q**_ and quasi-projection **k**_**q**_ are orthogonal. Also, it can be **k**_**q**_ = (0, 0, 1) (timelike). In our calculations, the quasi-projection vector will be chosen to be spacelike with value **k**_**q**_ = (0, 1, 0) or timelike with value **k**_**q**_ = (0, 0, 1), which gives the same value according to [22, 23].

In the case of *r*^*q*^ is spacelike curve, it has quasi-frame in the following form

$$\begin{array}{c} \frac{\partial }{\partial s}(\begin{array}{c} {\text{T}}_{q}\left(s\right)\\ {\text{n}}_{q}\left(s\right)\\ {\text{p}}_{q}\left(s\right) \end{array})=(\begin{array}{ccc} 0 & \tau_{1}\left(s\right) & -\tau_{2}\left(s\right)\\ -\tau_{1}\left(s\right) & 0 & \tau_{3}\left(s\right)\\ -\tau_{2}\left(s\right) & \tau_{3}\left(s\right) & 0 \end{array})(\begin{array}{c} {\text{T}}_{q}\left(s\right)\\ {\text{n}}_{q}\left(s\right)\\ {\text{p}}_{q}\left(s\right) \end{array}). \end{array}$$ (11)

The variation frame of *r*^*q*^ with respect to time can be written as

$$\begin{array}{c} \frac{\partial }{\partial t}(\begin{array}{c} {\text{T}}_{q}\\ {\text{n}}_{q}\\ {\text{p}}_{q} \end{array})=(\begin{array}{ccc} 0 & \sigma & \phi \\ -\sigma & 0 & \theta \\ \phi & \theta & 0 \end{array})(\begin{array}{c} {\text{T}}_{q}\\ {\text{n}}_{q}\\ {\text{p}}_{q} \end{array}), \end{array}$$ (12)

where *σ*, *ϕ* and *θ* are the velocities. For further information, we refer to [12, 14, 15, 20–24].

Specifically, we define the quasi-curvatures as

$$\begin{array}{c} \tau_{1}={\left\langle {\text{T}}_{q}^{\prime },{\text{n}}_{q}\right\rangle }_{E_{1}^{3}},\;\;\tau_{2}={\left\langle {\text{T}}_{q}^{\prime },{\text{p}}_{q}\right\rangle }_{E_{1}^{3}},\;\;\tau_{3}={\left\langle {\text{n}}_{q}^{\prime },{\text{p}}_{q}\right\rangle }_{E_{1}^{3}}. \end{array}$$ (13)

The relationship between quasi-frame and Frenet frame can be expressed as follows

$$\begin{array}{c} (\begin{array}{c} \text{T}\\ \text{n}\\ \text{p} \end{array})=(\begin{array}{ccc} 1 & 0 & 0\\ 0 & \text{cosh}(\zeta ) & \text{sinh}(\zeta )\\ 0 & -\text{sinh}(\zeta ) & -\text{cosh}(\zeta ) \end{array})(\begin{array}{c} {\text{T}}_{q}\\ {\text{n}}_{q}\\ {\text{p}}_{q} \end{array}), \end{array}$$ (14)

where *ζ* is the angle between **n** and **n**_*q*_. By means of *ζ*, the quasi-curvatures are read as

$$\begin{array}{c} \tau_{1}=\kappa cos\left(\zeta \right),\;\;\tau_{2}=-\kappa sin\left(\zeta \right),\;\;\tau_{3}=d\zeta +\tau {\left\langle {\text{n}}_{q}^{\prime },{\text{p}}_{q}\right\rangle }_{E_{1}^{3}}. \end{array}$$ (15)

We denote a q-frame for the frame {**T**_*q*_, **n**_*q*_, **p**_*q*_} when it is used for a q-spacelike curve. Also, the spacelike curve *r*^*q*^ in this paper will described as a spacelike q-curve that is accompanied by a timelike q-binormal.

## Evolution of a spacelike q-curve with timelike q-binormal

Our main finding in this phase of inquiry will be presented through the following theorems.

**Theorem 1** *For a given spacelike q-curve r^q^ with timelike q-binormal in the Minkowski space* $E_{1}^{3}$ , *the evolution equations of r^q^ via its q-frame can be described as*

$$\begin{array}{c} \left\{ \begin{array}{c} -\sigma_{s}-\phi \tau_{3}-\theta \tau_{2}=\tau_{1t},\\ -\phi_{s}-\sigma \tau_{3}+\tau_{1}\theta =\tau_{2t},\\ -\theta_{s}-\sigma \tau_{2}-\phi \tau_{1}=\tau_{3t}, \end{array}\right. \end{array}$$ (16)

*where σ, ϕ and θ are the velocities of the curve r*^*q*^.

**Proof**. We can write the q-frame of *r*^*q*^ given in (11) in a simple form

$$\begin{array}{c} \frac{\partial {\text{J}}_{\text{q}}}{\partial s}=L_{\text{q}}\;{\text{J}}_{\text{q}}, \end{array}$$ (17)

where

$$\begin{array}{c} {\text{J}}_{\text{q}}=(\begin{array}{c} {\text{T}}_{\text{q}}\\ {\text{n}}_{\text{q}}\\ {\text{p}}_{\text{q}} \end{array}),\;\;\;\;L_{\text{q}}=(\begin{array}{ccc} 0 & \tau_{1}\left(s\right) & -\tau_{2}\left(s\right)\\ -\tau_{1}\left(s\right) & 0 & \tau_{3}\left(s\right)\\ -\tau_{2}\left(s\right) & \tau_{3}\left(s\right) & 0 \end{array}). \end{array}$$

Also, Eq (12) can be arranged as follows:

$$\begin{array}{c} \frac{\partial {\text{J}}_{\text{q}}}{\partial t}={\text{S}}_{\text{q}}\;{\text{J}}_{\text{q}}, \end{array}$$ (18)

where

$$\begin{array}{c} {\text{S}}_{\text{q}}=(\begin{array}{ccc} 0 & \sigma (s,t) & \phi (s,t)\\ -\sigma (s,t) & 0 & \theta (s,t)\\ \phi (s,t) & \theta (s,t) & 0 \end{array}). \end{array}$$

By applying the compatibility conditions **J**_**q** *st*_ = **J**_**q** *ts*_ and making some calculations, one can get

$$\begin{array}{c} (\begin{array}{ccc} 0 & -\sigma_{s}-\phi \tau_{3}-\theta \tau_{2}-\tau_{1t} & -\phi_{s}-\sigma \tau_{3}+\tau_{1}\theta -\tau_{2t}\\ \sigma_{s}+\phi \tau_{3}+\theta \tau_{2}+\tau_{1t} & 0 & -\theta_{s}-\sigma \tau_{2}-\phi \tau_{1}+\tau_{3t}\\ -\phi_{s}-\sigma \tau_{3}+\tau_{1}\theta -\tau_{2t} & -\theta_{s}-\sigma \tau_{2}-\phi \tau_{1}+\tau_{3t} & 0 \end{array})=0_{3\times 3}, \end{array}$$ (19)

it leads to the required result.

Now, we will utilize the velocity vector of the q-curve under study to derive its evolution equations in another form.

**Theorem 2** *Let r^q^ be a spacelike q-curve which has q-frame* {**T**_*q*_, **n**_*q*_, **p**_*q*_} *with timelike q-binormal in Minkowski space* $E_{1}^{3}$. *Then, the evolution equations of r^q^ in terms of its q-velocity vector are*

$$\begin{array}{c} \frac{\partial }{\partial t}(\begin{array}{c} {\text{T}}_{q}\left(s,t\right)\\ {\text{n}}_{q}\left(s,t\right)\\ {\text{p}}_{q}\left(s,t\right) \end{array})=(\begin{array}{ccc} 0 & \frac{\alpha^{q}\tau_{1}+\beta_{s}^{q}+\gamma^{q}\tau_{3}}{c} & \frac{-\alpha^{q}\tau_{2}+\gamma_{s}^{q}+\beta^{q}\tau_{3}}{c}\\ \frac{-\alpha^{q}\tau_{1}-\beta_{s}^{q}-\gamma^{q}\tau_{3}}{c} & 0 & \theta (s,t)\\ \frac{-\alpha^{q}\tau_{2}+\gamma_{s}^{q}+\beta^{q}\tau_{3}}{c} & \theta (s,t) & 0 \end{array})(\begin{array}{c} {\text{T}}_{q}\left(s,t\right)\\ {\text{n}}_{q}\left(s,t\right)\\ {\text{p}}_{q}\left(s,t\right) \end{array}), \end{array}$$ (20)

where $\theta =\frac{1}{\tau_{1}}\left(-\tau_{2t}+{\left(\frac{\alpha^{q}\tau_{2}+\gamma_{s}^{q}+\beta^{q}\tau_{3}}{c}\right)}_{s}+\tau_{3}\left(\frac{\alpha^{q}\tau_{2}+\gamma_{s}^{q}-\beta^{q}\tau_{3}}{c}\right)\right)$.

**Proof**. We can write the flow of *r*^*q*^ as

$$\begin{array}{c} \frac{\partial r^{q}}{\partial t}=\alpha^{q}{\text{T}}_{q}+\beta^{q}{\text{n}}_{q}+\gamma^{q}{\text{p}}_{q}, \end{array}$$ (21)

where *α*^*q*^, *β*^*q*^ and *γ*^*q*^ are the q-velocities.

By differentiating Eq (21) with respect to *s*, one can obtain

$$\begin{array}{c} r_{ts}^{q}=\left(\alpha_{s}^{q}-\beta^{q}\tau_{1}-\gamma^{q}\tau_{2}\right){\text{T}}_{q}+\left(\alpha^{q}\tau_{1}+\beta_{s}^{q}+\gamma^{q}\tau_{3}\right){\text{n}}_{q}+\left(-\alpha^{q}\tau_{2}+\beta^{q}\tau_{3}+\gamma_{s}^{q}\right){\text{p}}_{q}. \end{array}$$ (22)

Since

$$\begin{array}{c} r_{s}^{q}=\left|\right|r_{s}^{q}\left|\right|{\text{T}}_{q}=c{\text{T}}_{q}, \end{array}$$ (23)

then, by differentiating Eq (23) with respect to *t* and using (12), we get

$$\begin{array}{c} r_{st}^{q}=\left|\right|r_{s}^{q}\left|\right|{\text{T}}_{qt}=c{\left(\sigma {\text{n}}_{q}+\phi {\text{p}}_{q}\right)}_{t}. \end{array}$$ (24)

Comparing the coefficients of q-tangent, normal, and binormal on both sides of Eqs (22) and (24), we have

$$\begin{array}{c} \left\{ \begin{array}{c} \sigma =\frac{\alpha^{q}\tau_{1}+\beta_{s}^{q}+\gamma^{q}\tau_{3}}{c}\\ \phi =\frac{-\alpha^{q}\tau_{2}+\gamma_{s}^{q}+\beta^{q}\tau_{3}}{c}\\ \theta =\frac{1}{\tau_{1}}\left(\tau_{2t}+{\left(\frac{\alpha^{q}\tau_{2}+\gamma_{s}^{q}+\beta^{q}\tau_{3}}{c}\right)}_{s}+\tau_{3}\left(\frac{-\alpha^{q}\tau_{2}+\gamma_{s}^{q}+\beta^{q}\tau_{3}}{c}\right)\right). \end{array}\right. \end{array}$$ (25)

Inserting the last equation in (12), the proof is completed.

## Geometry of Hasimoto surface and evolution of time

In this section, we interest with the evolution of a timelike Hasimoto surface generated by spacelike q-curve, so we give the following definition.

**Definition 1** *A surface in the Minkowski 3-space* $E_{1}^{3}$ , *is classified as spacelike or timelike based on whether the induced metric at the surface is a positive or negative definite Riemannian metric, respectively. Alternatively, it can be said that the normal vector on a spacelike surface is a timelike vector, while the normal vector on a timelike surface is a spacelike vector* [24].

Now, in light of the definition of Hasimoto surface, we will present and study the evolution of three types of Hasimoto surfaces by using the coefficients of their first and second fundamental forms. Also, we calculate the Gaussian and mean curvatures for these surfaces.

Let

$$\begin{array}{c} M^{q}:H^{q}=H^{q}\left(s,t\right), \end{array}$$ (26)

be the position vector of a generic point on timelike Hasimoto surface *M*^*q*^ in $E_{1}^{3}$, the vector

$$\begin{array}{c} {\text{N}}^{q}=\frac{H_{s}^{q}\times H_{t}^{q}}{\left|\right|H_{s}^{q}\times H_{t}^{q}\left|\right|}, \end{array}$$ (27)

determines the unit normal vector to *M*^*q*^ at the given point.

The first and second fundamental forms on *M*^*q*^ with their quantities are respectively, expressed by

$$\begin{array}{c} I=\left\langle dH^{q},dH^{q}\right\rangle =Eds^{2}+2\;Fdsdt+Gdt^{2}, \end{array}$$ (28)

where, $E=\langle H_{s}^{q},H_{s}^{q}\rangle ,\;F=\langle H_{s}^{q},H_{t}^{q}\rangle =\langle H_{t}^{q},H_{s}^{q}\rangle$, $G=\langle H_{t}^{q},H_{t}^{q}\rangle$, and

$$\begin{array}{c} II=\left\langle dH^{q},N^{q}\right\rangle =eds^{2}+2fdsdt+gdt^{2}, \end{array}$$ (29)

noting that $e=\langle H_{ss}^{q},N^{q}\rangle ,\;f=\langle H_{st}^{q},N^{q}\rangle =\langle H_{ts}^{q},N^{q}\rangle$ and $g=\langle H_{tt}^{q},N^{q}\rangle$.

The Gauss-Weingarten equations corresponding to the surface *M*^*q*^ give the rate of change of $\left(H_{s}^{q},H_{t}^{q},{\text{N}}^{q}\right)$ and take the following forms [13]

$$\begin{array}{c} \frac{\partial }{\partial s}(\begin{array}{c} H_{s}^{q}\\ H_{t}^{q}\\ N^{q} \end{array})=(\begin{array}{ccc} \Gamma_{11}^{1} & \Gamma_{11}^{2} & e\\ \Gamma_{21}^{1} & \Gamma_{21}^{2} & f\\ \frac{Ff-Ge}{\Delta } & \frac{Fe-Ef}{\Delta } & 0 \end{array})(\begin{array}{c} H_{s}^{q}\\ H_{t}^{q}\\ N^{q} \end{array}), \end{array}$$ (30)

$$\begin{array}{c} \frac{\partial }{\partial t}(\begin{array}{c} H_{s}^{q}\\ H_{t}^{q}\\ N^{q} \end{array})=(\begin{array}{ccc} \Gamma_{12}^{1} & \Gamma_{12}^{2} & F\\ \Gamma_{22}^{1} & \Gamma_{22}^{2} & G\\ \frac{Fg-Gf}{\Delta } & \frac{Fe-Eg}{\Delta } & 0 \end{array})(\begin{array}{c} H_{s}^{q}\\ H_{t}^{q}\\ N^{q} \end{array}). \end{array}$$ (31)

where Δ = *EG* − *F*^2^ and $\Gamma_{ij}^{k}$; *i*, *j*, *k* = 1, 2 are the quantities which are called Christoffel symbols of the second kind, for further details see [24, 25]. Here, the parameters $\Gamma_{ij}^{k}$ are

$$\begin{array}{c} \left\{ \begin{array}{c} \Gamma_{11}^{1}=\frac{1}{2\;\Delta }\left(GE_{s}-2FF_{s}+FE_{t}\right),\\ \Gamma_{12}^{1}=\Gamma_{21}^{1}=\frac{1}{2\;\Delta }\left(GE_{t}-FG_{s}\right),\\ \Gamma_{22}^{1}=\frac{1}{2\;\Delta }\left(-FG_{t}+2GF_{t}-GG_{s}\right),\\ \Gamma_{11}^{2}=\frac{1}{2\;\Delta }\left(-FE_{s}+2EF_{s}-EF_{t}\right),\\ \Gamma_{12}^{2}=\Gamma_{21}^{2}=\frac{1}{2\;\Delta }\left(EG_{s}-FE_{t}\right),\\ \Gamma_{22}^{2}=\frac{1}{2\;\Delta }\left(EG_{t}-2FF_{t}+FG_{s}\right). \end{array}\right. \end{array}$$ (32)

For more informations, please refer to [13, 25].

The Gaussian and mean curvatures *K*^*q*^, *H*^*q*^ are given by

$$\begin{array}{c} \left\{ \begin{array}{c} K^{q}=\epsilon_{N^{q}}\frac{Det(h)}{Det(\Delta )}\\ H^{q}=\frac{\epsilon_{N^{q}}}{2}\;\frac{eG-2fF+gE}{\left(EG-F^{2}\right)}=\frac{1}{2}\epsilon_{{\text{N}}^{q}}tr\left(h^{*}\Delta \right), \end{array}\right. \end{array}$$ (33)

where *h* = *eg* − *f*^2^, and h* denotes the inverse matrix of h and, *ϵ*_*N*^*q*^_ = 〈*N*^*q*^, *N*^*q*^〉 [24–26].

Differentiating (26) with regard to *s* and *t* and using Eqs (11), (12) and (14) yields

$$\begin{array}{c} \left\{ \begin{array}{c} H_{s}^{q}=c\;\text{T}=c\;{\text{T}}_{q},\\ H_{t}^{q}=\kappa \text{p}=\kappa \left(-\text{sinh}\left(\zeta \right){\text{n}}_{q}-\text{cosh}\left(\zeta \right){\text{p}}_{q}\right). \end{array}\right. \end{array}$$ (34)

More differentiating gives

$$\begin{array}{c} \left\{ \begin{array}{c} H_{ss}^{q}=c\left(\tau_{1}{\text{n}}_{q}-\tau_{2}{\text{p}}_{q}\right),\\ H_{tt}^{q}=\kappa \left(\sigma \;\text{sinh}\left(\zeta \right)-\phi \;\text{cosh}\left(\zeta \right)\right){\text{T}}_{q}-\left(\theta \kappa \;\text{cosh}\left(\zeta \right)+\kappa_{t}\;\text{sinh}\left(\zeta \right)\right){\text{n}}_{q}\\ \;\;\;\;\;\;-\left(\theta \kappa \;\text{sinh}\left(\zeta \right)+\kappa_{t}\;\text{cosh}\left(\zeta \right)\right){\text{p}}_{q}\\ H_{st}^{q}=H_{ts}^{q}=c\left(\sigma {\text{n}}_{q}+\phi {\text{p}}_{q}\right). \end{array}\right. \end{array}$$ (35)

Furthermore, the first and second fundamental forms with their coefficients are

$$\begin{array}{c} I=-c^{2}\kappa^{2}, \end{array}$$ (36)

$$\begin{array}{c} E=c^{2},\;\;\;\;\;F=0,\;\;\;\;\;and\;\;\;\;\;G=-\kappa^{2}, \end{array}$$ (37)

$$\begin{array}{c} II=-c\kappa_{t}\left(\tau_{1}\;\text{cosh}\left(\zeta \right)+\tau_{2}\;\text{sinh}\left(\zeta \right)\right)-c^{2}{\left(\phi \;\text{cosh}\left(\zeta \right)-\sigma \;\text{sinh}\left(\zeta \right)\right)}^{2} \end{array}$$ (38)

$$\begin{array}{c} e=-c\left(\tau_{1}\;\text{cosh}\left(\zeta \right)+\tau_{2}\;\text{sinh}\left(\zeta \right)\right),\;\;\;\;\;f=c\left(\phi \;\text{cosh}\left(\zeta \right)-\sigma \;\text{sinh}\left(\zeta \right)\right),\;\;\;\;\;and\;\;\;\;\;g=\kappa_{t}, \end{array}$$ (39)

Also, the Christoffel symbols are

$$\begin{array}{c} \Gamma_{22}^{1}=\frac{\kappa \kappa_{t}}{c^{2}},\;\;\;\;\;\Gamma_{12}^{2}=\Gamma_{21}^{2}=\frac{\kappa_{t}}{\kappa },\;\;\;\;\;\Gamma_{22}^{2}=\frac{\kappa_{t}}{\kappa }, \end{array}$$ (40)

as well as the others are determined for being zero.

In the light of this and using Gauss-Weingarten equations, the evolution of the first fundamental form coefficients are read

$$\begin{array}{c} \left\{ \begin{array}{c} \frac{\partial E}{\partial t}=-2\;e\;\gamma^{q}+2\;\alpha_{s}^{q}-2\;\Gamma_{11}^{1}\;\alpha^{q},\\ \frac{\partial F}{\partial t}=-2\;f\gamma^{q}+\beta_{s}^{q}-\Gamma_{12}^{1}\;\beta^{q}+\alpha_{t}^{q}-\Gamma_{12}^{1}\;\alpha^{q},\\ \frac{\partial G}{\partial t}=-2\;g\;\gamma^{q}+2\;\beta_{t}^{q}-2\;\Gamma_{22}^{2}\;\beta^{q}. \end{array}\right. \end{array}$$ (41)

According to the previous data, Eq (41) can be reformulated as

$$\begin{array}{c} \left\{ \begin{array}{c} \frac{\partial E}{\partial t}=-2c\left(\tau_{1}\;\text{cosh}\left(\zeta \right)+\tau_{2}\;\text{sinh}\left(\zeta \right)\right)\gamma^{q}+2\alpha_{s}^{q},\\ \frac{\partial F}{\partial t}=-2c\left(\phi \;\text{cosh}\left(\zeta \right)-\sigma \;\text{sinh}\left(\zeta \right)\right)\gamma^{q}+\alpha_{t}^{q}+\beta_{s}^{q}-\frac{\kappa_{t}}{\kappa }\alpha^{q},\\ \frac{\partial G}{\partial t}=-2\;\kappa_{s}\;\gamma^{q}+\alpha_{t}^{q}+2\left(\beta_{s}^{q}-\frac{\kappa_{s}}{\kappa }\beta^{q}\right). \end{array}\right. \end{array}$$ (42)

Similarly, the coefficients of the second fundamental form are expressed in the evolution form as

$$\begin{array}{c} \left\{ \begin{array}{c} \frac{\partial e}{\partial t}=\frac{1}{1+\Gamma_{11}^{2}\;\alpha^{q}}[\gamma_{ss}^{q}-\left(\Gamma_{11}^{1}\gamma_{s}^{q}+\Gamma_{11}^{2}\gamma_{t}^{q}\right)-e\;\left(E^{*}\;e+F^{*}\;g\right)\;\gamma^{q}+2\;e\;\left(\alpha_{s}^{q}-\Gamma_{11}^{1}\;\alpha_{s}^{q}-\Gamma_{11}^{2}\;\Omega_{t}\right)\\ \;\;\;\;\;\;\;\;+\alpha^{q}\;e_{s}\left(1-\Gamma_{11}^{1}\;\right)-f\;\left(E^{*}\;f+F^{*}\;g\right)\;\gamma^{q}+2\;f\;\beta_{s}^{q}\left(1-\Gamma_{11}^{1}\;\right)-\Gamma_{11}^{2}\;\beta_{t}^{q})\\ \;\;\;\;\;\;\;\;+\beta^{q}\left(f_{s}-\Gamma_{11}^{1}\;f_{s}-\Gamma_{11}^{2}\;f_{t}\right)],\\ \frac{\partial f}{\partial t}=\frac{1}{1-\beta^{q}\left(1-\Gamma_{22}^{2}\right)}\;[\gamma_{st}^{q}-\left(\Gamma_{12}^{1}\gamma_{s}^{q}+\Gamma_{12}^{2}\gamma_{t}^{q}\right)-e\;\left(G^{*}\;f+F^{*}\;e+\alpha_{t}^{q}-\Gamma_{12}^{1}\;\alpha_{s}^{q}-\Gamma_{12}^{2}\;\alpha_{t}^{q}\right)\\ \;\;\;\;\;\;\;\;+f\;\left(\alpha_{s}^{q}-\Gamma_{11}^{1}\alpha_{s}^{q}-\Gamma_{11}^{2}\alpha_{t}^{q}\right)+\alpha^{q}\;\left(e_{t}\left(1-\Gamma_{12}^{2}\right)-\Gamma_{21}^{1}e_{s}\right)-f\left(F^{*}\;f+G^{*}\;g\right)\gamma^{q}\\ \;\;\;\;\;\;\;\;+\beta_{t}^{q}\left(1-\Gamma_{22}^{2}\right)-\Gamma_{21}^{1}\;\beta_{s}^{q})+g\;\left(\beta_{s}^{q}\left(1-\Gamma_{11}^{1}\right)-\Gamma_{11}^{2}\;\beta_{t}^{q}\right)-\beta^{q}\Gamma_{22}^{2}\;f_{s}],\\ \frac{\partial g}{\partial t}=\frac{1}{1-\Gamma_{12}^{2}\;\beta^{q}}[\gamma_{tt}^{q}-\left(\Gamma_{22}^{1}\;\;\;\gamma_{s}^{q}+\Gamma_{22}^{2}\;\gamma_{t}^{q}\right)-f\;\left(F^{*}\;e+G^{*}\;f\right)\;\gamma^{q}\\ \;\;\;\;\;\;\;\;+2\;F\;\left(-\Gamma_{12}^{1}\;\alpha_{s}^{q}-\left(1-\Gamma_{12}^{2}\right)\;\alpha_{t}^{q}\right)+\alpha^{q}\;\left(f_{s}\left(1-\Gamma_{12}^{1}\right)-\Gamma_{12}^{2}\;f_{t}\right)\\ \;\;\;\;\;\;\;\;-2\;g[\left(F^{*}\;f+G^{*}\;g\right)\;\alpha^{q}-\Gamma_{12}^{1}\;\beta_{s}^{q}+\left(1-\Gamma_{12}^{2}\right)\;\beta_{t}^{q}]\\ \;\;\;\;\;\;\;\;+\beta^{q}g_{s}\left(1-\Gamma_{12}^{1}\right)], \end{array}\right. \end{array}$$ (43)

where e*, f* and g* refer to the inverse of *e*, *f* and *g* respectively.

In another word, we have

$$\begin{array}{c} \left\{ \begin{array}{c} \frac{\partial e}{\partial t}=\gamma_{ss}^{q}-{\left(\tau_{1}\;\text{cosh}\left(\zeta \right)+\tau_{2}\;\text{sinh}\left(\zeta \right)\right)}^{2}\;\gamma^{q}-2\;c\;\left(\tau_{1}\;\text{cosh}\left(\zeta \right)+\tau_{2}\;\text{sinh}\left(\zeta \right)\right)\;\alpha_{s}^{q}\\ \;\;\;\;\;\;\;\;\;-c\;\alpha^{q}\;{\left(\tau_{1}\;\text{cosh}\left(\zeta \right)+\tau_{2}\;\text{sinh}\left(\zeta \right)\right)}_{s}-{\left(\phi \;\text{cosh}\left(\zeta \right)-\sigma \;\text{sinh}\left(\zeta \right)\right)}^{2}\;\gamma^{q}\\ \;\;\;\;\;\;\;\;\;+2\;c\left(\phi \;\text{cosh}\left(\zeta \right)-\sigma \;\text{sinh}\left(\zeta \right)\right)\;\beta_{s}^{q}+c\;\beta^{q}\;{\left(\phi \;\text{cosh}\left(\zeta \right)-\sigma \;\text{sinh}\left(\zeta \right)\right)}_{s},\\ \frac{\partial f}{\partial t}=\frac{1}{1-\gamma^{q}\;\left(1-\frac{\kappa_{t}}{\kappa }\right)}\;[\gamma_{st}^{q}-\frac{\kappa_{t}}{\kappa }\;\gamma_{t}^{q}-\;\left(\tau_{1}\;\text{cosh}\left(\zeta \right)+\tau_{2}\;\text{sinh}\left(\zeta \right)\right)\;\left(\phi \;\text{cosh}\left(\zeta \right)-\sigma \;\text{sinh}\left(\zeta \right)\right)\;\gamma^{q}\\ \;\;\;\;\;\;\;\;-\left(\tau_{1}\;\text{cosh}\left(\zeta \right)+\tau_{2}\;\text{sinh}\left(\zeta \right)\right)\left(1-\frac{\kappa_{t}}{\kappa }\right)\;\;\alpha_{t}^{q}+c\left(\phi \;\text{cosh}\left(\zeta \right)-\sigma \;\text{sinh}\left(\zeta \right)\right)\;\alpha_{s}^{q}\\ \;\;\;\;\;\;\;\;\;-c\;\alpha^{q}\;\left({\left(\tau_{1}\;\text{cosh}\left(\zeta \right)+\tau_{2}\;\text{sinh}\left(\zeta \right)\right)}_{s}-\frac{\kappa_{t}}{\kappa }e_{t}\right)+\frac{1}{c}\;\left(\phi \;\text{cosh}\left(\zeta \right)-\sigma \;\text{sinh}\left(\zeta \right)\right)\;\kappa_{t}\;\gamma^{q}\\ \;\;\;\;\;\;\;\;\;+c\;\left(\phi \;\text{cosh}\left(\zeta \right)-\sigma \;\text{sinh}\left(\zeta \right)\right)\;\left(\beta_{t}^{q}\left(1-\frac{\kappa_{t}}{\kappa }\right)-\frac{\kappa \;\kappa_{t}}{a^{2}}\;\beta_{s}^{q}\right)+\kappa_{t}\;\beta_{s}^{q}\\ \;\;\;\;\;\;\;\;-c\;\beta^{q}\;\left(\frac{\kappa_{t}}{\kappa }\;{\left(\phi \;\text{cosh}\left(\zeta \right)-\sigma \;\text{sinh}\left(\zeta \right)\right)}_{s}\right)],\\ \frac{\partial g}{\partial t}=\frac{1}{1-\frac{\kappa_{t}}{\kappa }}[\gamma_{tt}^{q}-\frac{\kappa_{t}}{\kappa }\;\gamma_{t}^{q}+({\left(\tau_{1}\;\text{cosh}\left(\zeta \right)+\tau_{2}\;\text{sinh}\left(\zeta \right)\right)}^{2}\;\gamma^{q}\\ \;\;\;\;\;+2\;c\;\left(\left(\tau_{1}\;\text{cosh}\left(\zeta \right)+\tau_{2}\;\text{sinh}\left(\zeta \right)\right)\right)\;\left(1-\frac{\kappa_{t}}{\kappa }\right)\;\alpha_{t}^{q}+\alpha^{q}\;(c{\left(\left(\tau_{1}\;\text{cosh}\left(\zeta \right)+\tau_{2}\;\text{sinh}\left(\zeta \right)\right)\right)}_{s}\\ \;\;\;\;\;\;\;\;-\frac{\kappa_{t}}{\kappa }\;f_{t})-2\;\kappa_{t}\left(\left(-\frac{\kappa_{t}}{a^{2}}\right)\;\alpha^{q}+\;\beta_{t}^{q}\left(1-\frac{\kappa_{t}}{\kappa }\;\right)\right)+\beta^{q}{\left(\kappa_{t}\right)}_{s}]. \end{array}\right. \end{array}$$ (44)

Using Eq (33), the Gaussian and mean curvatures of *M*^*q*^ and their evolutions are respectively, given by

$$\begin{array}{c} \left\{ \begin{array}{c} K^{q}=\frac{-c\kappa_{s}\left(\tau_{1}\;\text{sinh}\left(\zeta \right)-\tau_{2}\;\text{cosh}\left(\zeta \right)\right)-c^{2}{\left(\phi \;\text{cosh}\left(\zeta \right)-\sigma \;\text{sinh}\left(\zeta \right)\right)}^{2}}{-c^{2}\kappa^{2}},\\ H^{q}=\frac{c^{2}\kappa_{t}+c\kappa^{2}\left(\tau_{1}\;\text{sinh}\left(\zeta \right)-\tau_{2}\;\text{cosh}\left(\zeta \right)\right)}{-c^{2}\kappa^{2}}, \end{array}\right. \end{array}$$ (45)

$$\begin{array}{c} \left\{ \begin{array}{c} \frac{\partial K^{q}}{\partial t}=\frac{\partial }{\partial t}\left(\frac{eg-f^{2}}{EG-F^{2}}\right),\\ \frac{\partial H^{q}}{\partial t}=\frac{\partial }{\partial t}\left(\frac{Ge-2fF+Eg}{2(EG-F^{2})}\right). \end{array}\right. \end{array}$$ (46)

### 0.1 Evolution of timelike Hasimoto surface attributed to the tangent of its q-curve

Now, we consider three types of Hasimoto surfaces generated by q-frame vectors of their spacelike q-curve *r*^*q*^ to study their geometric behavior and evolutions. For this, we present the following theorems.

**Theorem 3** *Let* $M^{q}:H^{q}=H^{q}\left(s,t\right)$ *be a timelike Hasimoto surface attributed to the q-tangent of a spacelike q-curve that has a timelike q-binormal. The surface M^q^ is an elliptic surface*.

**Proof**. Here, we can write Eq (26) as

$$\begin{array}{c} M^{q}:H^{q}\left(s,t\right)={\text{T}}_{q}\left(s,t\right). \end{array}$$ (47)

After differentiating (47) with respect to *s* and *t* and using Eq (27), we get

$$\begin{array}{c} {\text{N}}^{q}=\left(1,0,0\right). \end{array}$$ (48)

According to this, the first fundamental form coefficients are

$$\begin{array}{c} \left\{ \begin{array}{c} E=\left\langle H_{s}^{q},H_{s}^{q}\right\rangle =\tau_{1}^{2}-\tau_{2}^{2},\\ F=\left\langle H_{t}^{q},H_{s}^{q}\right\rangle =\left\langle H_{s}^{q},H_{t}^{q}\right\rangle =\tau_{1}\sigma +\tau_{2}\phi ,\\ G=\left\langle H_{t}^{q},H_{t}^{q}\right\rangle =\sigma^{2}-\phi^{2}, \end{array}\right. \end{array}$$ (49)

which lead to

$$\begin{array}{c} I=EG-F^{2}=\left(\tau_{1}^{2}-\tau_{2}^{2}\right)\left(\sigma^{2}-\phi^{2}\right)-{\left(\sigma \tau_{1}+\phi \tau_{2}\right)}^{2}. \end{array}$$ (50)

Also, the second fundamental form and its coefficients are, respectively

$$\begin{array}{c} II=eg-f^{2}=\left(-\tau_{1}^{2}+\tau_{2}^{2}\right)\left(-\sigma^{2}+\phi^{2}\right)-{\left(\sigma \tau_{1}+\phi \tau_{2}\right)}^{2}, \end{array}$$ (51)

and

$$\begin{array}{c} \left\{ \begin{array}{c} e=\left\langle H_{ss}^{q},N^{q}\right\rangle =-\tau_{1}^{2}+\tau_{2}^{2},\\ f=\left\langle H_{st}^{q},N^{q}\right\rangle =\left\langle H_{ts}^{q},N^{q}\right\rangle =-\left(\tau_{1}\sigma +\tau_{2}\phi \right),\\ g=\left\langle H_{tt}^{q},N^{q}\right\rangle =-\sigma^{2}+\phi^{2}. \end{array}\right. \end{array}$$ (52)

Besides, the Gaussian and mean curvatures are read

$$\begin{array}{c} \left\{ \begin{array}{c} K^{q}=1\\ H^{q}=-1. \end{array}\right. \end{array}$$ (53)

Since the surface *M*^*q*^ has constant values for its Gaussian and mean curvatures and the mean curvature is less than zero, then it is an elliptic surface.

### 0.2 Evolution of timelike Hasimoto surface attributed to the normal of its q-curve

**Theorem 4** *Assume that* $M^{q}:H^{q}=H^{q}\left(s,t\right)$ *be a timelike Hasimoto surface generated by the q- normal of spacelike q- curve that has a timelike q- binormal. The surface M^q^ is developable iff the following*

$$\begin{array}{c} \left(-\tau_{1}^{2}+\tau_{3}^{2}\right)\left(-\sigma^{2}+\theta^{2}\right)-{\left(-\tau_{1}\sigma +\tau_{3}\theta \right)}^{2}=0, \end{array}$$

*is satisfied*.

**Proof**. Write Eq (26) in the form

$$\begin{array}{c} M^{q}:H^{q}\left(s,t\right)={\text{n}}_{q}\left(s,t\right). \end{array}$$ (54)

If we differentiate (54) with respect to *s* and *t*, we obtain

$$\begin{array}{c} {\text{N}}^{q}=\left(0,1,0\right). \end{array}$$ (55)

We can get the first and second fundamental forms as follows

$$\begin{array}{c} I=\left(\tau_{1}^{2}-\tau_{3}^{2}\right)\left(\sigma^{2}-\theta^{2}\right)-{\left(\sigma \tau_{1}-\theta \tau_{3}\right)}^{2}, \end{array}$$ (56)

where

$$\begin{array}{c} E=\tau_{1}^{2}-\tau_{3}^{2},\;\;\;\;\;F=\tau_{1}\sigma -\tau_{3}\theta ,\;\;\;\;\;G=\sigma^{2}-\theta^{2}, \end{array}$$ (57)

and

$$\begin{array}{c} II=\left(-\tau_{1}^{2}+\tau_{3}^{2}\right)\left(-\sigma^{2}+\theta^{2}\right)-{\left(-\tau_{1}\sigma +\tau_{3}\theta \right)}^{2}, \end{array}$$ (58)

with notting that

$$\begin{array}{c} e=-\tau_{1}^{2}+\tau_{3}^{2},\;\;\;\;\;f=-\tau_{1}\sigma +\tau_{3}\theta ,\;\;\;\;\;g=-\sigma^{2}+\theta \phi . \end{array}$$ (59)

Also, from (33), we can obtain

$$\left\{\begin{array}{l} K^{q}=\frac{\left(-\tau_{1}^{2}+\tau_{3}^{2}\right)\left(-\sigma^{2}+\theta^{2}\right)-{\left(-\tau_{1}\sigma +\tau_{3}\theta \right)}^{2}}{\left(\tau_{1}^{2}-\tau_{3}^{2}\right)\left(\sigma^{2}-\theta^{2}\right)-{\left(\sigma \tau_{1}-\theta \tau_{3}\right)}^{2}},\\ H^{q}=\frac{\left(\tau_{1}^{2}-\tau_{3}^{2}\right)\left(-2\sigma^{2}+\theta \phi +\theta^{2}\right)-2\left(\tau_{1}\sigma -\tau_{3}\theta \right)}{2\left(\left(\tau_{1}^{2}-\tau_{3}^{2}\right)\left(\sigma^{2}-\theta^{2}\right)-{\left(\sigma \tau_{1}-\theta \tau_{3}\right)}^{2}\right)}. \end{array}\right.$$ (60)

As a result, the surface *M*^*q*^ is developable when

$$\begin{array}{c} \left(-\tau_{1}^{2}+\tau_{3}^{2}\right)\left(-\sigma^{2}+\theta^{2}\right)-{\left(-\tau_{1}\sigma +\tau_{3}\theta \right)}^{2}=0 \end{array}$$

Hence, the proof is completed.

### 0.3 Evolution of timelike Hasimoto surface attributed to the binormal to its q-curve

**Theorem 5** *Consider* $M^{q}:H^{q}=H^{q}\left(s,t\right)$ *be a timelike Hasimoto surface of a spacelike q-curve has a timelike q-binormal. The surface M^q^ is a hyperbolic surface*.

**Proof**. According to this case, Eq (26) can be put in the form

$$\begin{array}{c} M^{q}:H^{q}\left(s,t\right)={\text{p}}_{q}\left(s,t\right). \end{array}$$ (61)

After differentiating this equation with respect to *s* and *t*, we get the surface normal and have the following

$$\begin{array}{c} I=\left(\tau_{2}^{2}+\tau_{3}^{2}\right)\left(\phi^{2}+\theta^{2}\right)-{\left(-\phi \tau_{2}+\theta \tau_{3}\right)}^{2}, \end{array}$$ (62)

where

$$\begin{array}{c} E=\tau_{2}^{2}+\tau_{3}^{2},\;\;\;\;\;F=-\tau_{2}\phi +\tau_{3}\theta ,\;\;\;\;\;G=\phi^{2}+\theta^{2}. \end{array}$$ (63)

Also, we obtain

$$\begin{array}{c} II=\left(-\tau_{1}\tau_{2}+\tau_{3s}\right)\left(\phi \sigma -\theta_{t}\right)-{\left(\tau_{1}\phi +\theta_{s}\right)}^{2}, \end{array}$$ (64)

with

$$\begin{array}{c} e=\tau_{2}^{2}+\tau_{3}^{2},\;\;\;\;\;f=-\phi \;\tau_{2}+\theta \;\tau_{3},\;\;\;\;\;g=\phi^{2}+\theta^{2}. \end{array}$$ (65)

After using Eq (33), the Gaussian and mean curvatures are

$$\begin{array}{c} K_{q}=-1\;\;\;\;\;H_{q}=-1 \end{array}$$ (66)

From this, the evolved surface *M*^*q*^ is classified as a hyperbolic surface.

## Application

In this section, we provide an example that illustrates the evolution of Hasimoto surface of the timelike type to show the theoretical findings of this paper.

Let $M^{q}:H^{q}=H^{q}\left(s,t\right)$ be a timelike Hasimoto surface given with a parametric representation

$$\begin{array}{c} H^{q}\left(s,t\right)=(s-2\;tanh(s),-2\;sech(s)\;sinh(t),-2sech(s)\text{cosh}(t)), \end{array}$$

and consider

$$\begin{array}{c} r^{q}\left(s\right)=\left(s-2\;tanh\left(s\right),0,-2\;sech\left(s\right)\right), \end{array}$$

be its spacelike q-curve, then *κ* and *τ* are given by

$$\begin{array}{c} \kappa =2\;sech(s),\;\tau =0. \end{array}$$ (67)

The q-frame **T**_*q*_, **n**_*q*_, **p**_*q*_ are calculated as

$$\begin{array}{c} \left\{ \begin{array}{c} {\text{T}}_{q}=\left(1-2\;sech^{2}\left(s\right),0,2\;sech\left(s\right)\;tanh\left(s\right)\right),\\ {\text{n}}_{q}=\left(-2\;cosh\left(\zeta \right)\;sech\left(s\right)\text{tanh}\left(s\right),-\text{sinh}\left(\zeta \right),2\;cosh\left(\zeta \right)\left(1-2\;sech^{2}\left(s\right)\right)\right),\\ {\text{p}}_{q}=\left(2\;\text{sinh}\left(\zeta \right)\;sech\left(s\right)\;\text{tanh}\;\left(s\right),\text{cosh}\left(\zeta \right),2\;\text{sinh}\left(\zeta \right)\left(-1+2\;sech^{2}\left(s\right)\right)\right). \end{array}\right. \end{array}$$ (68)

Also, the curvatures of the q-curve are given by

$$\begin{array}{c} \tau_{1}=-2\;sech\left(s\right)\text{cosh}\left(\zeta \right),\;\;\;\;\;\tau_{2}=2\;sech\left(s\right)\text{sinh}\left(\zeta \right),\;\;\;\;\;\tau_{3}=0. \end{array}$$ (69)

The coefficients of the first fundamental form are

$$\begin{array}{c} E=1,\;\;\;\;\;F=0,\;\;\;\;\;G=-4\;sech^{2}\left(s\right), \end{array}$$

which lead to

$$\begin{array}{c} \Delta =-4\;sech^{2}\left(s\right). \end{array}$$

Besides, the surface normal is

$$\begin{array}{c} N^{q}=\left(2\;sech\left(s\right)\;\text{tanh}\;\left(s\right),\left(-1+2\;sech{\left(s\right)}^{2}\right)\text{sinh}\left(t\right),\left(-1+2\;sech{\left(s\right)}^{2}\right)\text{cosh}\left(t\right)\right), \end{array}$$ (70)

and the coefficients of the second fundamental form are

$$\begin{array}{c} e=2\;sech\left(s\right),\;\;\;\;\;f=0,\;\;\;\;\;g=\;sech^{3}\left(s\right)\left(\text{cosh}\left(2s\right)-3\right), \end{array}$$

where

$$\begin{array}{c} h=2\;sech^{4}\left(s\right)\left(\text{cosh}\left(2s\right)-3\right). \end{array}$$

From which, we have the Gaussian and mean curvatures as follows

$$\begin{array}{c} \left\{ \begin{array}{c} K^{q}=\frac{1}{2}\left(3-\text{cosh}\left(2s\right)\right)\;sech^{2}\left(s\right),\\ H^{q}=\frac{1}{8}\left(11-\text{cosh}\left(2s\right)\right)\;sech\left(s\right)). \end{array}\right. \end{array}$$ (71)

**Fig 1:**
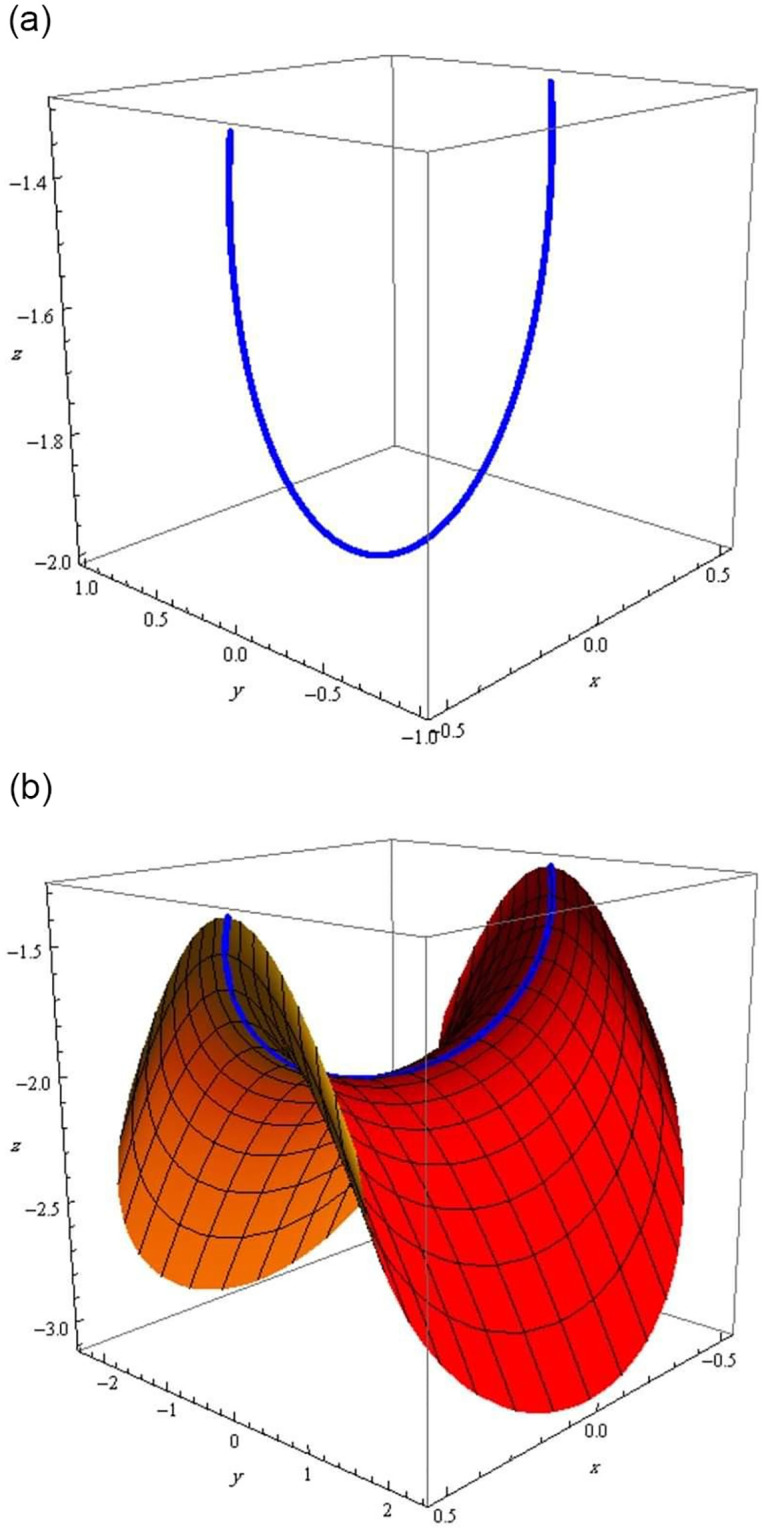
The spacelike q-curve *r*^*q*^ and timelike Hasimoto surface $H^{q}$. (a) q-curve *r*^*q*^, (b) Hasimoto surface $H^{q}$.

The evolved curve and its timelike Hasimoto surface are shown respectively, in Fig 1a and 1b.

**Fig 2:**
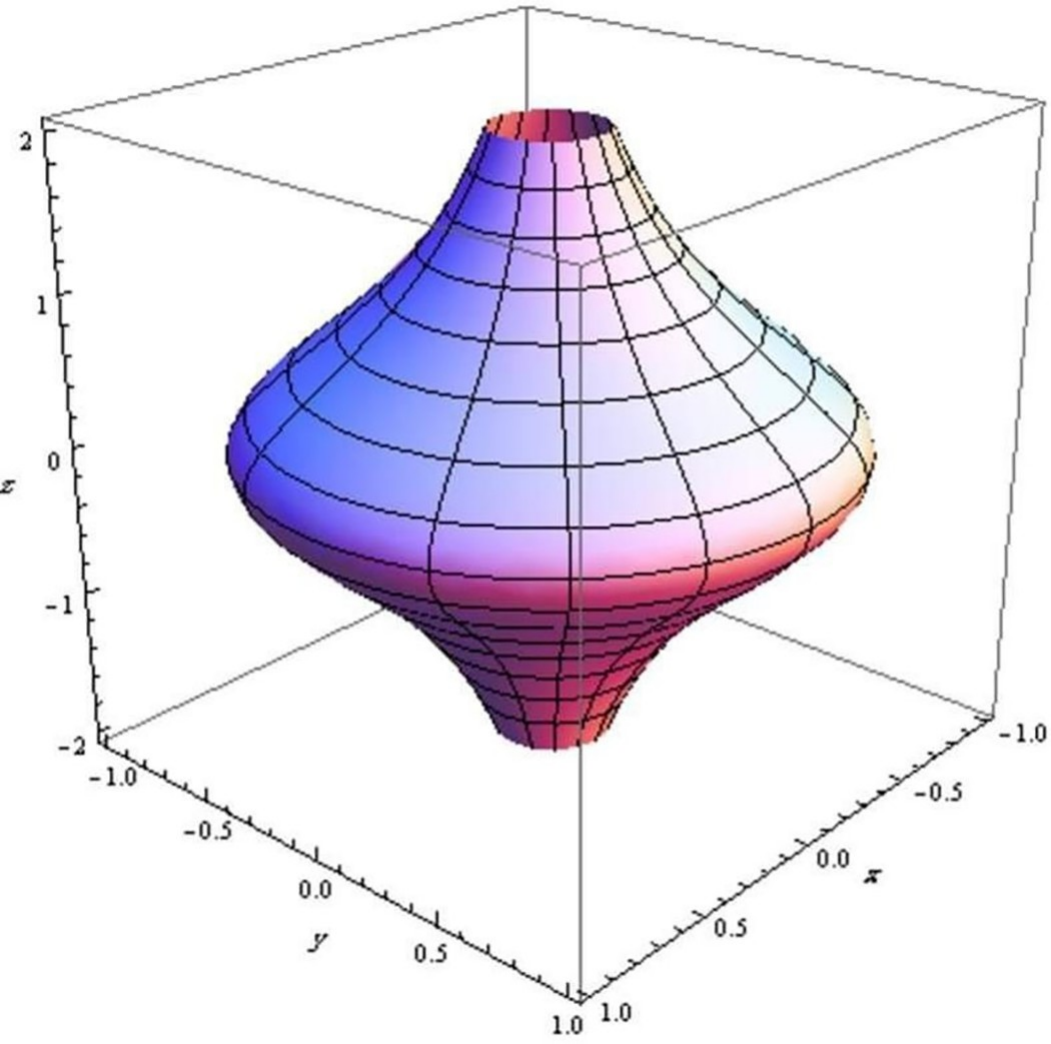
The evolution of an elliptic surface with respect to q-curve’s tangent.

**Fig 3:**
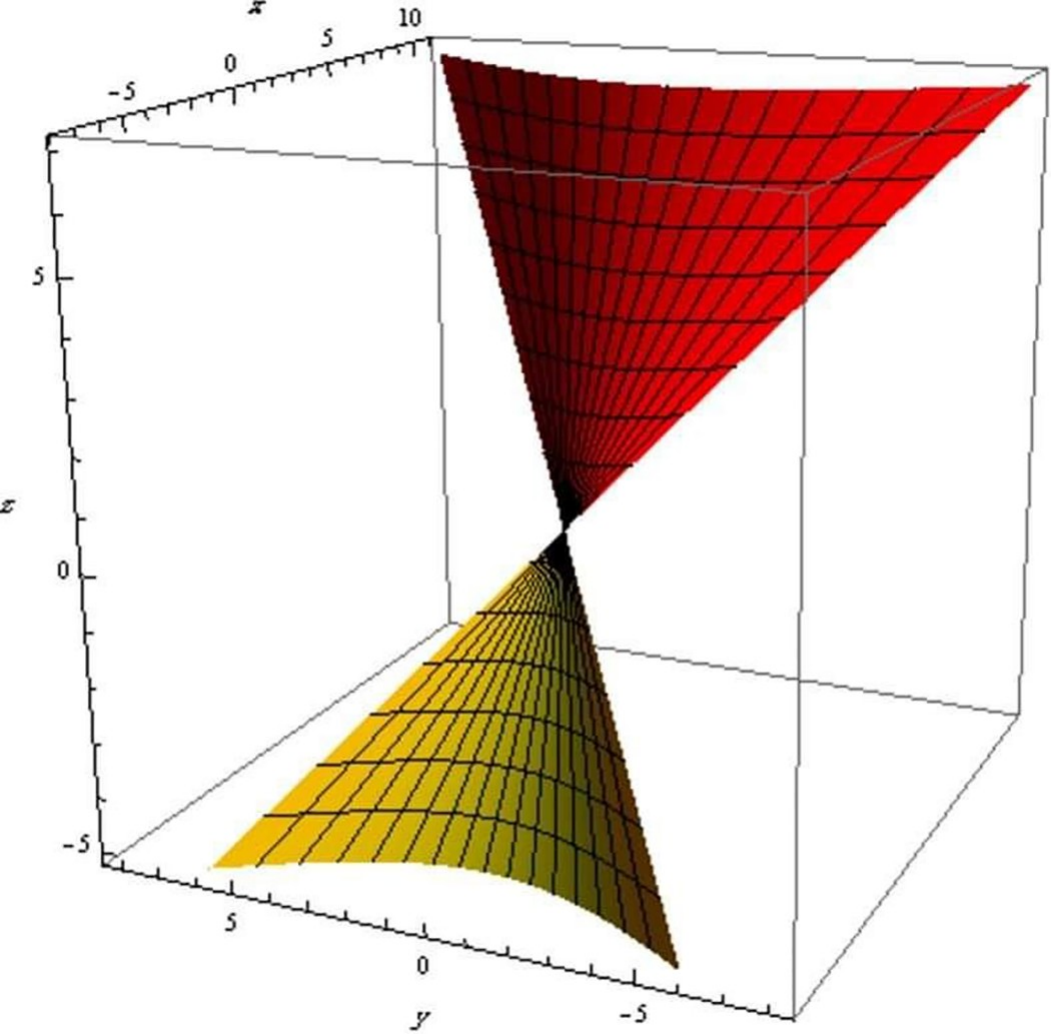
The evolution of a developable surface with respect to q-curve’s normal.

**Fig 4:**
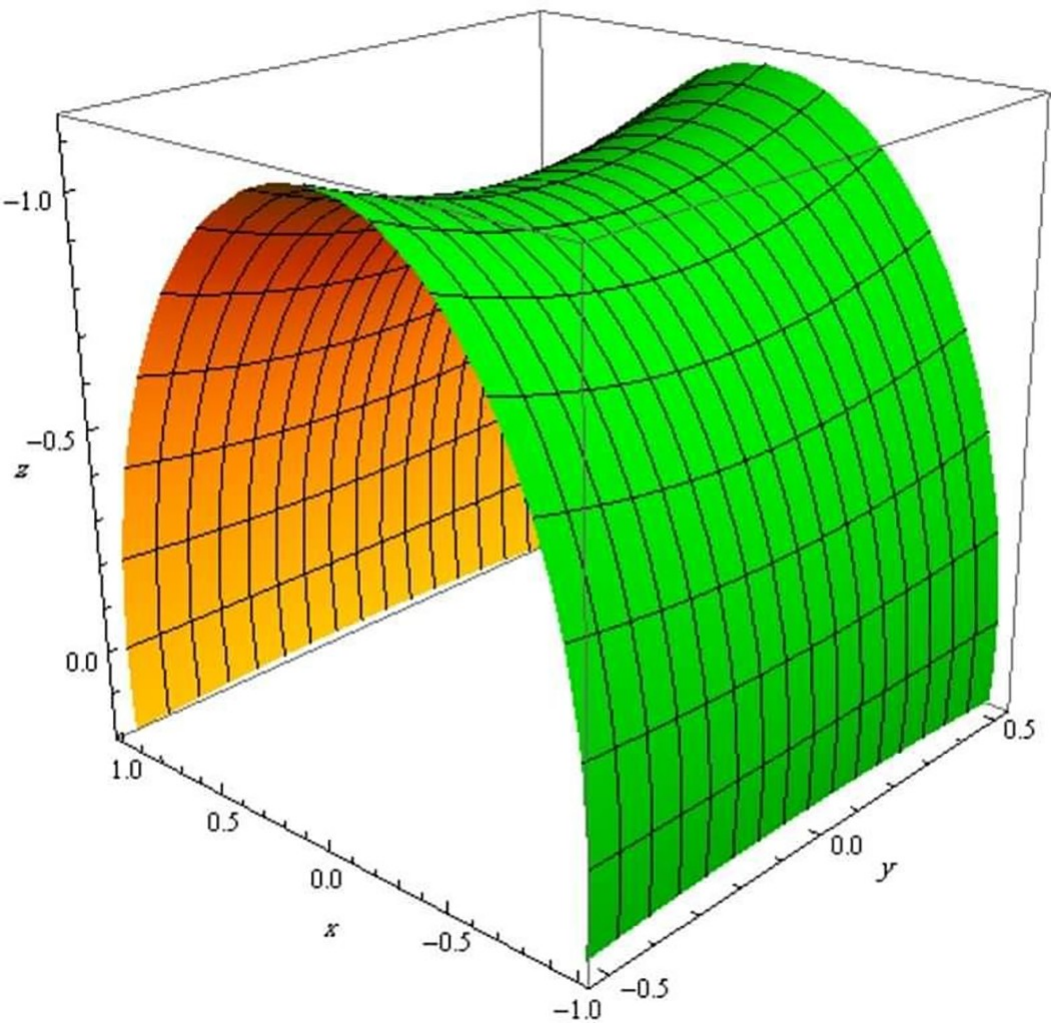
The evolution of a hyperbolic surface with respect to q-curve’s binormal.

Finally, we show the evolution of three surfaces with respect to the q-frame vectors in Figs 2–4.

## Conclusion

The evolution equations and some geometric properties for a timelike Hasimoto surface in Minkowski 3-space have been introduced. For this purpose, Gauss and Weingarten equations as well as the evolution of the evolved q-curve associated to the considered Hasimoto surface have been used. In addition, the evolution for the coefficients of the first and second fundamental forms and the Gaussian and mean curvatures for the surface have been determined. Moreover, three types of the evolved surface have been presented and analyzed. As a consequence, it is noted that some values of the Gaussian and mean curvatures for these surfaces are constants whereas the others depending on the velocities of the evolved q-curve. Finally, a computational example to illustrate our main results has been given and plotted.

In future work, we plan to investigate the harmonic evolute surfaces of the Hasimoto surface in different spaces, including Galilean and pseudo-Galilean spaces. We aim to enhance the results presented in this paper by incorporating techniques and findings from related studies [27–37]. Additionally, we intend to explore novel methods to discover further results and theorems concerning the singularity and symmetry properties of this topic, which will be presented in our upcoming papers. This endeavor underscores the significance and potential future developments of these surfaces.
